# Postveraison Deficit Irrigation Effects on Fruit Quality and Yield of “Flame Seedless” Table Grape Cultivated under Greenhouse and Net

**DOI:** 10.3390/plants9111437

**Published:** 2020-10-25

**Authors:** Virginia Pinillos, Sonia Ibáñez, Jéssica M. Cunha, Juan J. Hueso, Julián Cuevas

**Affiliations:** 1 Departamento de Agronomía, ceiA3, Universidad de Almería, 04120 Almería, Spain; sib468@inlumine.ual.es (S.I.); jcuevas@ual.es (J.C.); 2 Setor de Nutrição Mineral de Plantas, Universidade Estadual do Norte Fluminense Darcy Ribeiro, RJ 28013-602 Campos dos Goytacazes, Brazil; jessimcunha@yahoo.com.br; 3 Estación Experimental de Cajamar Las Palmerillas, Paraje Las Palmerillas, El Ejido, 04710 Almería, Spain; juanjosehueso@fundacioncajamar.com

**Keywords:** *Vitis vinifera*, water stress, protected cultivation, berry size, yield, fruit earliness

## Abstract

Lack of color in the skin of red table grape varieties is a serious problem in areas of warm climate. This problem is often addressed by the application of ethylene release products such as ethephon. Strict regulation in the use of this product in EU forces European grape producers to look for suitable alternatives. With the aim to increase red skin color, we applied regulated deficit irrigation (RDI) strategies from veraison until harvest on “Flame Seedless” table grape vines cultivated under nets and under a plastic greenhouse in South East Spain, and compared yield and fruit quality with vines fully irrigated under the same net and plastic greenhouses. Our results show a modest improvement in the percentage of commercial clusters with better skin color, probably because the short duration of the deficit irrigation period only caused a slight decrease in soil water content and a mild water stress in RDI vines. Larger differences were observed under the more limiting conditions of the plastic greenhouse for light environment, especially when berry skin color was measured by CIRG (color index of red grape). More noticeable effect of RDI was noted on fruit earliness. Water savings were also remarkable. Negative effects of RDI on berry size or total soluble solid content were not perceived. Our results suggest that RDI is a suitable strategy to save irrigation water without substantial negative effects on yield and berry size. However, the effects on skin color were insufficient in the trial conditions.

## 1. Introduction

Intensity and homogeneity of berry skin color is one of the main quality attributes in red table grapes. Unfortunately, in warm climate regions, red table grapes often present color deficits that decrease their commercial value [1,2]. Poor color has been related to high temperatures during ripening since they seem to inhibit the accumulation of anthocyanins in the skin of the berry and prevent color development [3,4]. Anthocyanins synthesis can be affected by many different factors, among them, temperature, light intensity, nutrition, and irrigation [5]. Some of these factors are strongly modified by the use of covering materials in protected cultivation (either under greenhouse or net), thus aggravating poor color development in the skin of the fruit [6]. Cultural practices common in table grape, such as leaf removal and shoot and cluster thinning, seek enhancing berry appearance [7,8], but commonly are not enough to resolve color deficits. On the contrary, other practices intended to increase berry size, such as the application of gibberellic acid, inhibit or delay color acquisition [9,10].

Applications of ethephon (2-chloroethylphosphonic acid), an ethylene-releasing compound, after veraison improve skin color and have been used extensively by table grape growers. Abscisic acid (ABA) applications are also effective for improving skin color in grapes [1]. ABA partially regulates the synthesis and accumulation of anthocyanins in the skin of the fruit [11,12,13] and its exogenous application significantly increases pigment content in the skin of the grapes [14]. In different red grape cultivars, ABA treatments induced also a more rapid coloring of the berry skin anticipating harvest and increasing the amount of marketable yield, without negative effects on sugar and acid content [9,14]. Some cultivars, such as “Crimson Seedless”, need just one application of ABA [10], but others like “Niagara Rosada” and “Rubí” require several applications [15,16]. In warm areas, two applications of ABA are often needed [2]. Despite their efficiency, the application of ABA and ethephon may affect berry firmness reducing its quality and postharvest storage period [14]. Besides that, current European laws have drastically reduced maximum residues levels of ethephon in fruits, which limits its use in table grapes.

Regulated deficit irrigation (RDI) may be an alternative to the use of plant growth regulators to improve berry color in grape, since one of the responses of vines to drought is the increase of ABA synthesis [17,18,19,20,21]. ABA content in berries nearly doubled in response to water stress in the “Cabernet Sauvignon” wine grape cultivar, allowing an increase in anthocyanins in the fruits [11]. In seedless table grapes, better berry color and earlier ripening have been observed in vines subjected to RDI from veraison to harvest [22]. Pinillos et al. [23] found that postveraison RDI induced earlier harvest in “Crimson Seedless” due to an earlier acquisition of berry color in RDI vines. These authors explained the lack of negative effect of RDI on berry size by the short duration of the deficit irrigation period, nonetheless long enough for affecting positively skin color in “Crimson Seedless”. Conesa et al. [24], working also with “Crimson Seedless”, showed that it is possible to reduce irrigation by RDI, without negatively affecting yield or quality. They also found that RDI increased some bioactive compounds, such as the antioxidant resveratrol, beneficial for human health.

The objective of this study was to determine the effect of RDI in yield and berry quality of “Flame Seedless” (especially berry skin color and size) grapevines grown under different protected cultivation conditions (plastic greenhouse versus net).

## 2. Material and Methods

### 2.1. Experimental Site and Vine Management

The experiments were carried out in two consecutive years (2015 and 2016) on “Flame Seedless” vines grafted on 161-49 Couderc. “Flame Seedless” is a seedless early variety, with high commercial value, that normally ripens around mid-July in the southeast coastal area of Spain. Earliness is enhanced in this variety by greenhouse production, a cultivation system that clearly increases its profitability [25]. Rootstock 161-49 Couderc is tolerant to calcareous soils and shows mid to high resistance to drought. 161-49 Couderc seems to aggravate color acquisition in the varieties grafted on it. In this regard, higher values of greenness and lower of redness have been found in berries of verities grafted on 161-49 Couderc [26].

**Figure 1:**
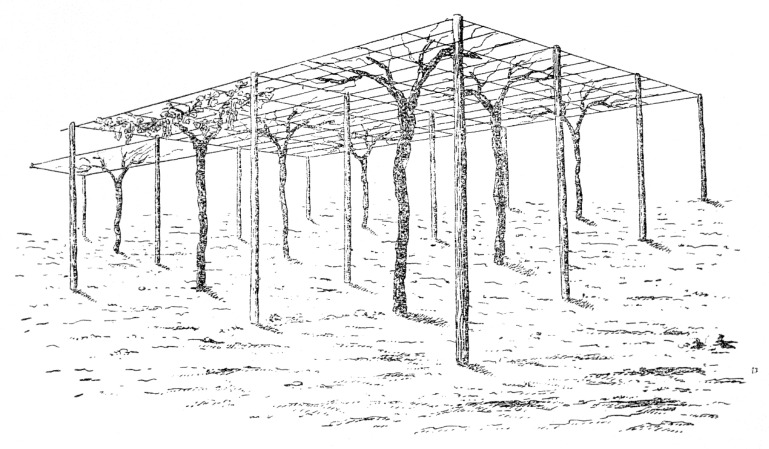
The Spanish parral training system showing vines, structure and wires used to support canopy and crop load.

The experimental vines were planted in 1999 in an experimental orchard of the Cajamar Foundation sited in El Ejido (Almería, Spain) (long. 2°43’10” W; lat. 36°47’40” N; alt. 151 m). Experimental vines were spaced at 3.5 × 3.5 m. As usual in the area, the experimental plants were trained following a trellis system known as Spanish parral, consisting in a single trunk approximately divided in four main scaffold branches at 2.10 m height. The productive wood (canes) are directly inserted into the main branches (Figure 1).

According to the classification of Papadakis, the climate of the area is semiarid subtropical Mediterranean. The annual average temperature is close to 18.5 °C. The coolest months are December and January, while the warmest month is August. Rainfall in the site is very scarce, only around 250 mm per year. The mean relative humidity ranges between 67 and 73% along the season. Very frequent sunny days make that sunlight hours reach an average of 3273 per year. The soil of the orchard is a sandy clay loam with a proportion of 49.6% of sand, 26.4% of silt and 24.0% of clay, at 10–70 cm depth, where most roots of the vines grow.

Crop load of the experimental vines was carefully adjusted by pruning and thinning to avoid initial differences in crop load to interfere with the response of the plants to deficit irrigation and color acquisition. Pruning, carried out in February before bud break, consisted in leaving, in each experimental vine, 10 canes (productive wood) of eight buds each and several spurs of 23 buds for replacement. Crop load was further adjusted by cluster thinning, leaving 70 clusters per vine. Gibberellic acid (GA3) was applied 23 times during flowering at 10 ppm to favor flower thinning and reduce the number of berries per cluster. A second treatment consisting in an application of GA3 at the dose of 50 ppm was performed when the fruit reached a diameter of 67 mm, and repeated the following week at the same dose (five and four weeks before veraison). This application aims to enlarge remaining berries.

Fertilizers were applied through the irrigation system at a seasonal rate of 80–60–175 kg ha^−1^ of N, P_2_O_5_ and K_2_O, respectively. The fertilization program did not vary between treatments and plant management did not differ in any other regards. The plants were maintained free of pest and diseases by means of conventional practices included in integrated pest guidelines.

### 2.2. Experimental Design and Measurements

Two experiments were performed following a completely randomized design. The first experiment was carried out with vines covered with a polyethylene net of 6 × 6 wires per cm^2^ and 66.4% porosity. The second experiment replicated the former but was executed in vines enclosed under a greenhouse covered with a clear low-density polyethylene film 200 microns thick. Natural ventilation in the plastic greenhouse was achieved by four zenithal windows oriented east–west, and two lateral windows oriented north–south. The opening of the windows was regulated by a Priva weather controller with a temperature set point adjusted of 15 °C from bud swelling to the beginning of flowering and 25 °C from flowering to fruiting.

The same two irrigation treatments were established in each environment: a control treatment, where the vines received 100% of their water needs throughout the season, and a regulated deficit irrigation treatment (RDI), where the vines only received 25% of the irrigation needs from the beginning of veraison to the end of harvest. Three replicates per treatment, each one composed of four vines, were established. RDI vines received full irrigation from budbreak (first days of February) to the beginning of veraison (commencement of RDI period) and from the end of harvest until the end of November, when the irrigation ended in both treatments. Therefore, the irrigation treatments differed only in the water applied between the beginning of veraison and the end of harvest. In this period, control vines were irrigated four times a week, while RDI vines only once a week, so the irrigation treatments differed in the number of irrigations per week, but not in the volume of water being applied per irrigation operation.

The water needs of the vines were determined according to an estimation of crop evapotranspiration (ET_c_ = ET_o_ × K_c_). ET_o_ was calculated using two US class A pan evaporimeters located in the same orchard (one in the open air and one inside a plastic greenhouse), while the crop coefficients were locally adjusted for both cultivation system taking into account the recommendations made for early-season seedless tables grapes cultivars grown in southeast Spain [27] and our experience managing different table grape vineyards in the experimental site (Table 1).

The beginning of veraison, the start of the RDI treatment, was determined by carefully monitoring the phenological stage of the berries, and was considered as the moment in which most experimental vines had more than 50% of the clusters with some colored berries. Based on these observations, the beginning of veraison was established in 2015 as 12 June and 30 June, in the greenhouse and under net, respectively, and as 10 June and 29 June in 2016 in the plastic greenhouse and under net, respectively.

Soil water content was measured by time domain reflectometry (TDR) (model 6050 × 1 Trase System I, Soil Moisture Equipment Co., Santa Barbara, CA, USA), just before and after the implementation of the irrigation treatments and every week during the RDI period. Two waveguides of 45 cm length were installed in each replicate. Topp’s equation was used to estimate soil water content [28]. Plant water status in response to the irrigation treatments was monitored by measuring midday stem water potential (Ψ_st_ with a pressure chamber (model 3000, Soil Moisture Co., Santa Barbara, CA, USA) at the same dates of soil water content measurements, using three adult healthy north-facing leaves per replication (one per vine). Leaves were bagged and covered with aluminum foil for two to three hours before measurements carried out between 13:00 and 14:00 h [29].

We compared total yield per plant (kg per vine) and earliness (by mean of harvest date, and by the number and dates of picking and the volume of yield harvested in each picking operation). Harvest started when the total soluble solid content (TSS) in samples composed of the most advanced berries (picking is based on skin color) reached 15° Brix. For this determination, 15 berries per vine were weekly collected from veraison until beginning of harvest. Harvest was performed weekly, collecting only clusters with commercial color. In each harvest operation, the collected clusters were classified according to the percentage of well-colored berries: clusters with more than 80% of colored berries and clusters with between 60% and 80% of colored berries. In the last harvest operation, all clusters were picked regardless of their color, that is, non-commercial cluster with less than 60% of colored berries were also harvested.

**Figure 2:**
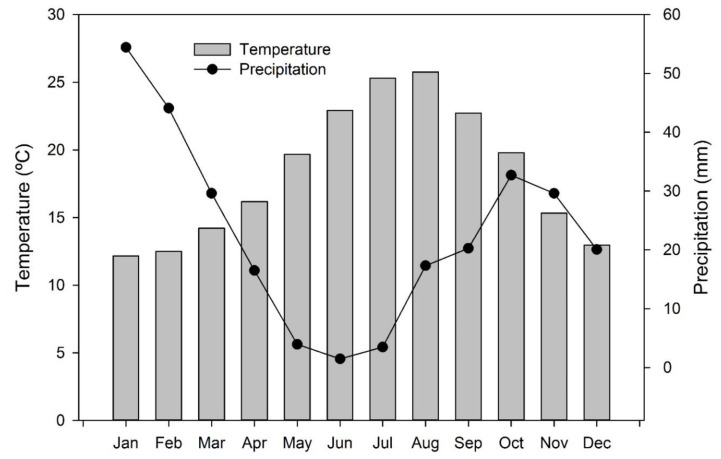
Average monthly temperature (line) and rainfall (bars) based on a 17-year period (2000–2016). Source: La Mojonera Agroclimactic Station (Junta de Andalucía).

Water use efficiency (WUE) was calculated as the total yield divided by the irrigation water applied plus rain fallen during the whole season. Rainfall was ignored for vines grown in the greenhouse. Rainfall in season 2015 was 220.2 mm, while in 2016 it was 192.2 mm. The monthly distributions of rainfall and mean temperatures of each season are shown in Figure 2.

Berry size (weight and diameter) and quality (TSS; titratable acidity, TA, and skin color) were compared using samples of 20 berries per vine (60 berries per replicate) collected in the most representative picking operation of each treatment (the heaviest one). TSS was measured using a digital refractometer (model PAL-1, Atago Co., Tokyo, Japan). TA was determined by titration with 0.1 N NaOH using phenolphthalein as indicator and expressed as g tartaric acid L^−1^. Berry skin color was measured using a colorimeter (model CR-400, Konica Minolta Co., Osaka, Japan), and represented in the CIE-Lab space. Hue (h°), Chroma and color index of red grape (CIRG) indexes were calculated from L*, a*, and b* values as recommended by Carreño et al. [30]. CIRG was also determined in random samples of berries (20 berries per vine) of every picking operation in order to follow changes in skin color through the harvest season.

Irrigation treatments were compared in both scenario by Student’s *t* tests using Statistix 8.0 software.

## 3. Results

### 3.1. Irrigation Water Applied

RDI reduced irrigation water consumption in percentages between 15% and 26% depending on the year and cultivation system (Table 2). As it was expected, greenhouse cultivation did reduce water consumption compared to the net due to the lower evapotranspiration taking place in the former. This occurred in the whole growing season as well as during the period in which deficit irrigation was imposed. Actual water savings during the RDI period fluctuated between 72.6% and 77.0%, close to the estimated saving of 75% programmed for this phase (Table 2). The differences in the irrigation water applied to vines grown in the greenhouse versus vines grown under net were also remarkable (about 33% in both years), favoring the former. The amount of irrigation water applied was very similar in both seasons.

### 3.2. Soil Water Content

**Figure 3:**
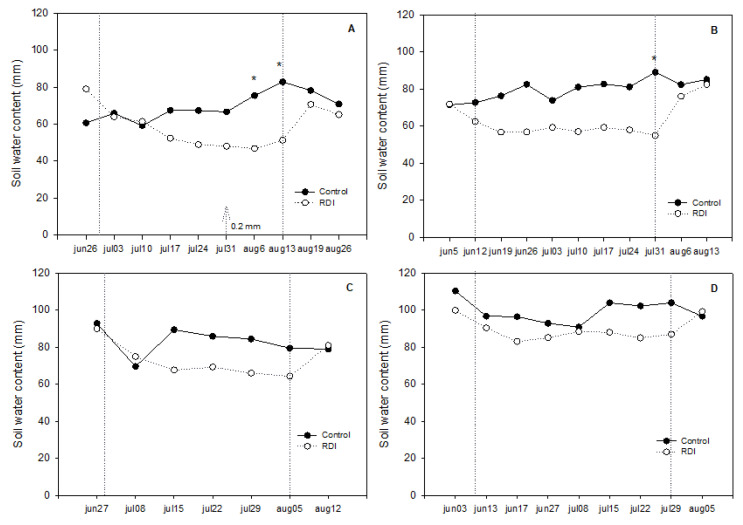
Soil water content at 45 cm depth in control and regulated deficit irrigated (RDI) vines cultivated under net (**left**, **A** and **C**) and under a plastic greenhouse (**right**, **B** and **D**) in 2015 (**top**) and 2016 (**bottom**). Vertical lines mark deficit irrigation periods. Arrows represent the rain events during the deficit irrigation period. When present, * indicates significant differences between irrigation treatments (*p* < 0.05).

The reduction of water applied during DI period led to a progressive diminution of soil water content around RDI plants (Figure 3). However, the differences between irrigation treatments reached statistical significance only in 2015, in the last weeks of the experiment just before irrigation was fully restarted (Figure 3A,B). Soil water content did not vary around control plants, while it showed an initial decline, the first weeks after water restriction, in RDI replicates (Figure 3A–D). In RDI plants under net, soil water content fell near 45 mm at the end of the RDI period in 2015, while in control vines soil moisture was between 60 and 70 mm (Figure 3A). In the greenhouse, the lowest value observed in RDI plants was below 60 mm during the DI period, but around 80 mm in control plants (Figure 3B), suggesting better conditions for plants growing under plastic. Once irrigation was restarted all plants reached similar values, confirming that the irrigation scheduling was correct.

Similar trend was observed in 2016, although soil water content was higher in both cultivation system in this year, and significant differences between irrigation treatments were never reached. Resuming irrigation after harvest made again soil moisture levels to come to equal values in both irrigation treatments (Figure 3C,D).

### 3.3. Stem Water Potential

**Figure 4:**
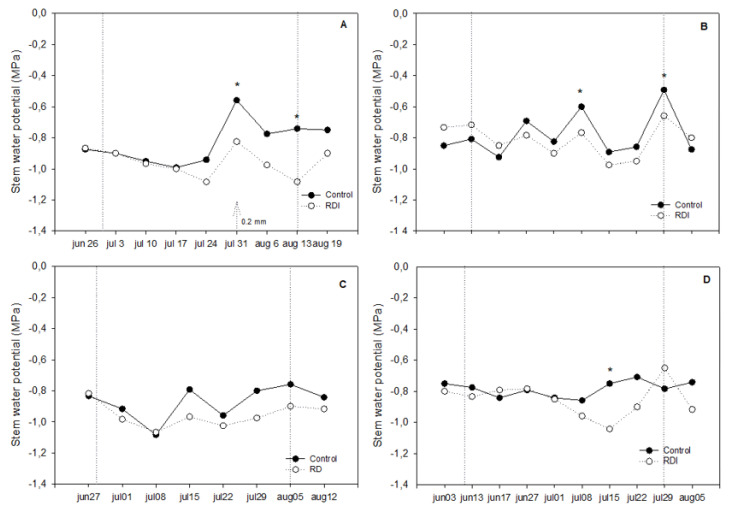
Stem water potential in control and regulated deficit irrigated (RDI) vines cultivated under net (**left**, **A** and **C**) and under a plastic greenhouse (**right**, **B** and **D**) in 2015 (**top**) and 2016 (**bottom**). Vertical lines mark deficit irrigation periods. Arrows represent the rain events during the deficit irrigation period. When present, * indicate significant differences between irrigation treatments (*p* < 0.05).

In spite of the significant reduction in soil water content observed in 2015, plants subjected to RDI only showed a mild level of water stress as measured Ψ_st_ values suggest. In this regard, in 2015, the most negative values were slightly above and below −1.0 MP, depending on cultivation system (Figure 4A,B). Significant differences between treatments were only observed at the end of the RDI period. Similar trends were observed in 2016, with significant differences between irrigation treatments only under plastic greenhouse at the end of the RDI period (Figure 4D). In any case, Ψ_st_ values of both irrigation treatments were quite similar in the greenhouse as well as under net in both seasons (Figure 4A–D).

It was not unusual to observe abrupt changes in Ψ_st_ values from one week to the next. This occurred in plants protected by net and by plastic cover. Explanations for these changes are not obvious, although slight showers and cloudy days could be behind them. Nonetheless, we recorded a steady decline in Ψ_st_ values along the DI period in RDI vines, while control vines gave more stable values, especially in 2016 (Figure 4A–D).

Finally, Ψ_st_ values were slightly more negative under net responding to the above reported lower soil moisture observed in this cultivation system and suggesting again better conditions for plants grown under the greenhouse.

### 3.4. Yield

RDI significantly reduced total yield in the vines cultivated in the plastic greenhouse in both seasons (Table 3). Under this condition, yield in RDI vines was reduced between 21% and 33% with respect to control vines. On the contrary, no significant differences between irrigation treatments were observed under net in any year (Table 3). Differences in yield between cultivation systems were small in both years. In 2015, average yield under mesh was slightly higher than in the greenhouse (26.7 ± 1.7 vs. 25.8 ± 2.8 kg per vine), but on the contrary, in 2016, greenhouse production was a little more (29.2 ± 1.9 vs. 28.8 ± 2.3 kg per vine in greenhouse and net, respectively). Average cluster weight oscillated between 310 g and 470 g. No significant differences were found in this regard between irrigation treatments in any of the protected environments and years. In general, average cluster tended to be higher in the treatments with higher yield (Table 3).

**Figure 5:**
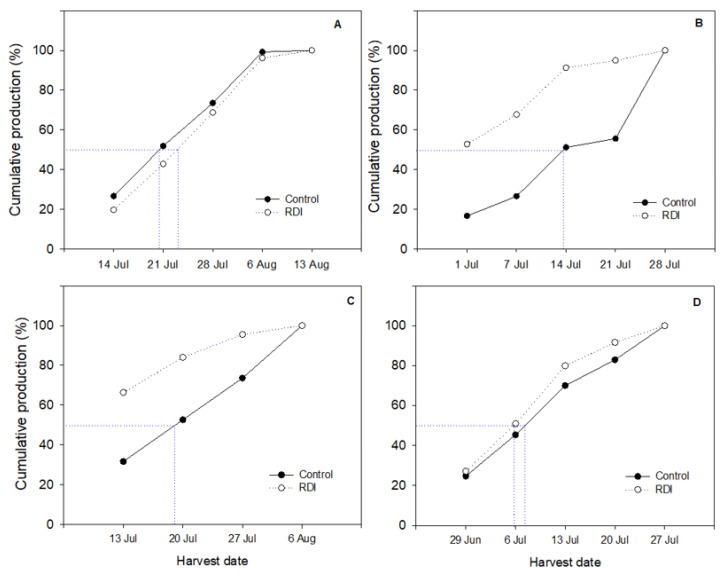
Cumulative production in control versus regulated deficit irrigated (RDI) “Flame Seedless” vines grown under net (**A** and **C**) and plastic greenhouse (**B** and **D**) in 2015 (**top**) and in 2016 (**bottom**).

Harvest started earlier in the greenhouse in both seasons as expected (Figure 5). In 2015, harvest was initiated on 1 July, while in 2016 it started at 29 June. Under net, harvest began two weeks later in both seasons. Harvest was also more intense in RDI vines: the first picking date had a heavier yield, especially in greenhouse in 2015 and under net in 2016. In these cases, the production picked from RDI vines in the two first harvesting times doubled the production harvested in control vines (Figure 5).

Due to difficulties in the development of a nice skin color, especially at the late harvest, some poor-colored clusters were finally picked. For this reason, just after picking, the clusters were classified in two main categories depending on their color (well-colored clusters, with more than 80% of berries with nice color and non-well-colored clusters, with only 60–80% of colored berries). Production of well-colored clusters was not significantly different between irrigation treatments in any condition and year, but in general its proportion was higher in RDI vines (Table 4). In RDI vines, well colored clusters represented between 81% and 91% of total yield, while in control vines well colored clusters were between 41% and 88%. Contrarily, non-well-colored cluster production was higher in control vines (where it represented between 8% and 39% of total yield) than in RDI vines (with values representing between 5% and 18% of total yield); these differences between treatments were statistically significant in the greenhouse. Likewise, the production of the noncommercial clusters (those with less than 60% of colored berries) that were collected in the last harvest date was also higher in control plants.

### 3.5. Water Use Efficiency (WUE)

RDI had a variable effect on WUE. In 2015, the less amount of water applied besides the higher yield increased WUE in RDI plants under net. However, the reduced yield of RDI plants under greenhouse led to a smaller WUE compared to control plants despite the water savings achieved (Table 4). On the contrary, in 2016, in spite of the lower yield in RDI vines, the reduction in the water applied increased WUE with respect to control vines in greenhouse, although this reduction was not high enough to improve WUE in vines grown under net (Table 4).

### 3.6. Fruit Quality

Despite the differences between treatments in the harvesting rhythm, the most representative picking operation can be considered the same in both treatments and cultivation systems, and similar in both seasons (July 14 and July 13 in 2015 and 2016, respectively). No significant differences between RDI and control vines were found in any of the quality parameters in this most representative harvest time in any season (Table 5). However, slightly bigger and especially heavier fruits were harvested from control vines although differences were not significant.

On the other hand, the berries were larger and heavier in vines grown in greenhouses compared to vines under net. TSS content was higher in vines grown under net in 2015, but no difference was found in 2016 between cultivation systems. Fruit acidity seemed lower in the greenhouse both years.

Regarding skin color, irrigation treatment did not have significant effect on any parameters. The only exception was hue values in the greenhouse in the second year (Table 6), when berries from RDI vines showed a significant lower h° value (17.6 vs. 24.8), meaning a more intense red color. CIRG was also higher (more reddish) in RDI berries in this growing condition in 2016, although the difference between irrigation treatments were just close to statistical significance (*p* = 0.06).

**Figure 6:**
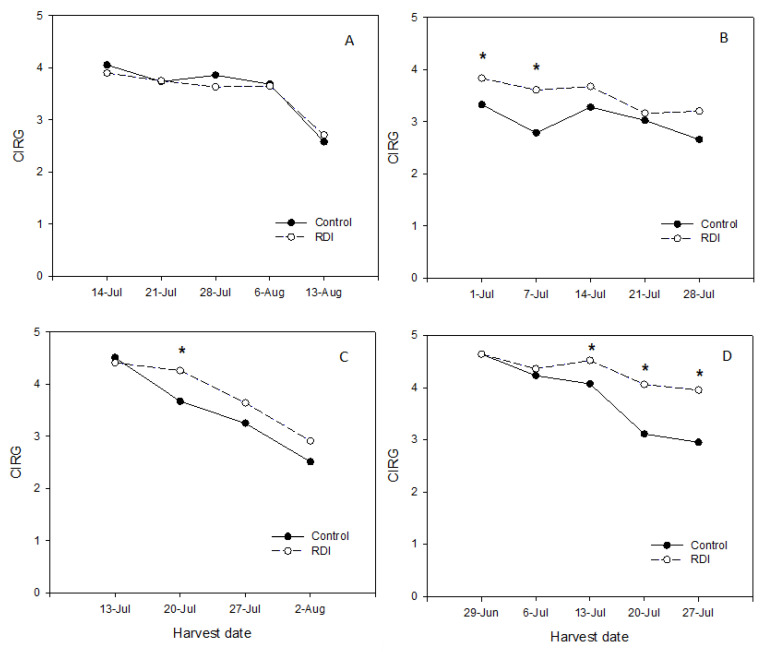
Evolution of the red grape color index (CIRG) throughout the harvest season in plants cultivated under net (**A** and **C**) and under a plastic greenhouse (**B** and **D**) in 2015 (**top**) and 2016 (**bottom**). When * is present, it indicates significant differences between irrigation treatments (Student’s T test, *p* < 0.05).

In general, the berries presented poorer color in the skin as the harvest season progressed from July to August. In this sense, CIRG average values in greenhouse decreased from mean values of 3.6 and 4.6 at the first picking time to 2.9 and 3.5 in the last one, in 2015 and 2016, respectively (Figure 6). Under net, mean CIRG values decreased from 4.0 and 4.5 to 2.7 and 2.8, in 2015 and 2016 as harvest season advanced (Figure 6). In general, CIRG values were higher in berries from RDI vines than in control vines throughout the harvest season, and in some picking dates these differences were statistically significant (although not, as above explained, in the most representative harvest) (Figure 6).

## 4. Discussion

RDI during ripening has shown promising results, enhancing red skin color in several wine and table grape cultivars [22,24,31,32]. In fact, previous results in experiments carried out by us on “Crimson Seedless” (a midseason cultivar) in the same location showed earlier and more intense acquisition of red color in the skin of the berries leading to higher commercial yield, without negative effects on berry size or production [23].

In the case of “Flame Seedless”, our results show that berry skin color improvement due to postveraison RDI was modest (Table 6). Although the results seemed better when expressed in percentage of clusters showing nice color (Table 3), the true is that the improvement achieved by RDI was insufficient for growers to adopt this as a solution for poor color in “Flame Seedless” berries. Better results for the CIRG index (in comparison to controls) were obtained in vines cultivated under the plastic greenhouse (Table 6), where conditions of high temperature worsen berry color achievement. We think that berry color improvement was limited because the RDI period was too short and water stress too mild with significant differences in plant water status appearing too late in the season (Figure 4). This in turn seems related to the slight decrease in water soil content measured in response to irrigation withholding that rarely reached significant differences, and, when it did, it was again too late in the season (Figure 3).

On the other hand, a more severe RDI treatment is expected to exert a more negative effect on berry size in a variety falling short of size very often. Wise balance between negative RDI effects on berry size and positive on color might force to perform additional berry thinning within the clusters, thus further reducing cluster weight and yield. As stated before, better results were obtained by our team on “Crimson Seedless” (with longer periods of RDI). Several authors have also documented color improvement in wine and table grape cultivars in response to DI [22,24,31,33].

Although, RDI only slightly improved the color of the berry, water savings were noticeable (Table 2). The results show that, as expected, postveraison RDI reduced water consumption (23% in the greenhouse and 18% under net) with respect to control vines (Table 3). Soil water content around deficit irrigated plants diminished as the period of RDI progressed causing significant differences with respect to control in the last dates (Figure 3). However, this diminution in soil water content did not cause any severe water stress. On the contrary, the effects were mild and only significant in two measurements late in the season once harvest had already started (Figure 4). In this regard, the minimum values of midday Ψ_st_ measured in response to the reduction in soil water content only descended a little below −1.0 MPa, threshold value considered as nonstressful in grape [33].

Due to the mild effects of RDI on plant water status, no significant differences in berry diameter or weight were caused. Although a common response to RDI is a yield reduction accompanied of smaller and lighter berries [34,35,36], these effects depend on the level, duration and phenological stage in which RDI is applied. RDI during fruit set and enlargement is clearly prejudicial for berry size at harvest, but if water stress takes place late in berry development, once veraison has started, then the diameter of the berry remains largely unaffected [37,38,39]. Although not significantly, negative effects of RDI on fruit weight were more pronounced in 2016 (Table 5) in both greenhouse and net cultivation systems. As a consequence of reducing berry weight, the clusters formed in RDI vines were lighter (Table 3), reducing yield in some amount (significantly under greenhouse) (Table 3). As it has been said before, yield reduction in response to water stress is a common feature in deficit irrigated vines, although if DI is imposed after veraison yield can be largely unaffected [22,24,40], as occurred here in vines grown under net. Pinillos et al. [23] noted an improvement in water use efficiency under RDI in “Crimson Seedless” thanks to the water savings achieved without negative effects on yield. Here, on the contrary, water use efficiency was not always improved, because yield losses attributable to RDI were more often greater, especially under greenhouse conditions (Table 4).

Finally, protected cultivation under plastic greenhouse enhanced early maturity in “Flame Seedless” regardless of irrigation treatments, reaching mean harvest date two weeks earlier than under net. This advancement allows more profitable harvest date as previously demonstrated on different table grape cultivars [7,25,41]. Earliness was even more enhanced if RDI was applied, allowing thus a higher proportion of clusters to be harvested at the first dates (Figure 5). Earliness in response to preharvest RDI is a common feature observed in different fruit crops [42,43], table grape included [23].

## 5. Conclusions

Postveraison RDI slightly improved berry skin color especially in vines cultivated under plastic, where RDI also enhanced earliness but caused a significant reduction in yield due to the production of lighter berries. Effects on vines cultivated under net were smaller and rarely significant. Despite important water savings, this trial showed that RDI as applied in these two experiments was not able to solve the lack of color found in warm areas of southeast Spain.

## Figures and Tables

**Table 1:** Crop coefficients (Kc) and evapotranspiration (ET_o_) (mm) for net and greenhouse cropping systems.

**Net**												
	**Jan**	**Feb**	**Mar**	**Apr**	**May**	**Jun**	**Jul**	**Aug**	**Sep**	**Oct**	**Nov**	**Dec**
K_c_^a^	-	0.28	0.30	0.33	0.40	0.45	0.45	0.38	0.30	0.30	0.25	-
ET_o_^b^	47	64	90	113	140	164	200	165	116	87	56	45
**Greenhouse**												
K_c_^a^	--	0.30	0.30	0.38	0.40	0.43	0.40	0.35	0.3	0.30	0.25	-
ET_o_^b^	31	29	37	60	101	113	121	106	82	57	37	27

^a^ Kc locally adjusted. ^b^ ET_o_ average measured in the last 10 years in the site.

**Table 2:** Total water applied (TWA) throughout the season, water applied during the deficit irrigation period (WA-RDI) in both seasons and cultivation system. Between parentheses, in TWA column, annual water savings (%) in RDI plants with respect to control plants.

		2015		2016	
	Treatment	TWA (mm)	WA-RDI (mm)	TWA (mm)	WA-RDI (mm)
Net	Control	475.0	112.0	473.0	94.6
	RDI	389.2 (18.1%)	26.2 (76.6%)	400.2 (15.4%)	21.8 (77.0%)
Greenhouse	Control	317.1	96.0	315.0	114.9
	RDI	244.3 (23.0%)	23.0 (76.0%)	231.6 (26.5%)	31.5 (72.6%)

**Table 3:** Yield in response to irrigation treatments (Control and regulated deficit irrigation (RDI)) in vines grown under net and under a plastic greenhouse, in the 2015 and 2016 seasons. Cluster type: well-colored cluster, with more than 80% well colored berries (>80%); non-well-colored cluster, with 60–80% well colored berries (60–80%); and noncommercial clusters (harvested only in the last picking operation) (<60%).

2015						2016				
Treatments	Total Yield (kg Vine^−1^)	Yield Per Cluster Type (kg Vine^−1^)			Cluster Weight (kg)	Total Yield (kg Vine^−1^)	Yield Per Cluster Type (kg Vine^-1^)			Cluster Weight (kg)
		>80%	60–80%	<60%			>80%	60–80%	<60%	
			**Net**					**Net**		
Control	24.9	22.0	2.0	0.4	0.35	31.6	21.8	8.2	1.5	0.46
RDI	28.6	24.3	3.9	0.4	0.39	26.0	22.9	2.8	0.4	0.37
*p*	n.s.	n.s	n.s	n.s.	n.s	n.s.	n.s.	n.s.	n.s.	n.s.
			**Greenhouse**					**Greenhouse**		
Control	31.1 a *	12.6	12.1 a	5.9 a	0.38	33.1 a	26.0	4.6 a	1.3	0.41
RDI	20.6 b	16.6	3.8 b	0.2 b	0.31	26.0 b	23.6	1.3 b	0.2	0.36
*p*	0.04	n.s	0.01	0.03	n.s		n.s	0.03	n.s	n.s

* In columns, values followed by different letters are significantly different (n.s: non-significant; Student’s *t* test *p* < 0.05)

**Table 4:** Water use efficiency (WUE) for the irrigation treatments in vines grown under net and under plastic greenhouse, in 2015 and 2016. (n.s.: nonsignificant, Student’s *t* test *p* < 0.05)**.**

	2015	2016
Treatment	WUE (kg m^−3^)	WUE (kg m^−3^)
	**Net**	
Control	2.9	3.9
RDI	3.8	3.6
*p*	n.s.	n.s.
	**Greenhouse**	
Control	8.0	8.6
RDI	6.9	9.2
*p*	n.s.	n.s.

**Table 5:** Berry quality parameters in the most representative picking operation in response to irrigation treatments (Control and regulated deficit irrigation (RDI)) in vines grown under net and plastic greenhouse in 2015 and 2016. (n.s.: nonsignificant, Student’s *t* test *p* < 0.05)**.**

	2015				2016			
Treatments	Diameter (cm)	Weight (g)	TSS (°Brix)	TA (g L^−1^ Tartaric Acid)	Diameter (cm)	Weight (g)	TSS (°Brix)	TAn(g L^−1^ Tartaric Acid)
	**Net**				**Net**			
Control	1.8	3.6	16.2	5.5	1.8	3.6	14.9	4.0
RDI	1.8	3.5	16.2	6.1	1.7	3.0	15.3	4.6
*p*	n.s	n.s	n.s	n.s	n.s	n.s	n.s	n.s
	**Greenhouse**				**Greenhouse**			
Control	1.9	4.2	14.9	5.0	1.9	4.7	15.4	2.0
RDI	1.9	4.1	15.0	4.8	1.8	4.2	15.9	2.1
*p*	n.s	n.s	n.s	n.s	n.s	n.s	n.s	n.s

**Table 6:** Color parameters (Lightness (L*), chroma (C*), hue (h°) and CIRG) of ’Flame Seedless’ berries in the most representative picking operation in response to irrigation treatments (control and regulated deficit irrigation (RDI) in vines cultivated under net and greenhouse in 2015 and 2016. * in columns, values followed by different letters are significantly different (Student’s *t* test *p* < 0.05).

	2015				2016			
**Treatment**	**Net**							
	L*	C *	**h°**	**CIRG**	L*	C*	**h°**	**CIRG**
Control	31.4	8.2	37.7	4.1	29.9	6.9	47.0	4.4
RDI	32.1	9.3	44.8	3.9	29.5	7.5	32.3	4.5
*p*	0.35	0.32	0.71	0.44	0.41	0.42	0.31	0.85
	**Greenhouse**							
	L*	C*	**h°**	**CIRG**	L*	C*	**h°**	**CIRG**
Control	33.1	9.4	52.0	3.3	30.9	7.9	24.8 a	4.1
RDI	32.3	8.6	46.4	3.7	30.1	6.6	17.6 b	4.5
*p*	0.30	0.55	0.55	0.19	0.04	0.20	0.04	0.06
